# Study protocol: The efficacy of mushroom to prevent cognitive decline in at-risk middle-aged adults and young-olds living in the community

**DOI:** 10.3389/fnagi.2025.1588493

**Published:** 2025-06-09

**Authors:** Jiatong Shan, Luwen Cao, Jiuyu Guo, Kai Xuan Lim, Na Li, Yinxia Chao, Yizhen Xie, Jian Chen, Wei Ma, Su Lin Lim, Irwin Kee-Mun Cheah, Mihir Gandhi, Tih-Shih Lee, Lei Feng, Kaisy Xinhong Ye

**Affiliations:** ^1^Centre for Healthy Longevity, @AgeSingapore, National University Health System, Singapore, Singapore; ^2^Department of Psychological Medicine, Yong Loo Lin School of Medicine, National University of Singapore, Singapore, Singapore; ^3^Healthy Longevity Translational Research Program, Yong Loo Lin School of Medicine, National University of Singapore, Singapore, Singapore; ^4^Jinlin Ginseng Academy, Changchun University of Chinese Medicine, Changchun, China; ^5^Duke-NUS Medical School, Singapore, Singapore; ^6^Department of Neurology, Singapore General Hospital, National Neuroscience Institute, Singapore, Singapore; ^7^Guangdong Provincial Key Laboratory of Microbial Safety and Health, State Key Laboratory of Applied Microbiology Southern China, Institute of Microbiology, Guangdong Academy of Sciences, Guangzhou, China; ^8^Guangdong Yuewei Edible Fungi Technology Co., Ltd., Guangzhou, China; ^9^Department of Geriatric Respiratory Medicine, Guangzhou First People's Hospital, South China University of Technology, Guangzhou, China; ^10^Department of Dietetics, National University Hospital, Singapore, Singapore; ^11^Department of Biostatistics, Singapore Clinical Research Institute, Singapore, Singapore; ^12^Centre for Quantitative Medicine, Duke-NUS Medical School, Singapore, Singapore; ^13^Duke-NUS Signature Research Programme in Neuroscience and Behavioural Disorders, Singapore, Singapore

**Keywords:** mushroom, cognitive decline, RCT, cohort study, dementia prevention

## Abstract

**Background:**

Cognitive function declines with increasing age and maintaining high cognitive functioning especially at late life remains a challenging question to be addressed. Emerging evidence in the role of mushroom in promoting cognition has been produced from limited observational studies but there is a lack of definitive evidence on both longitudinal relationships from prospective cohort studies and clinical efficacy from clinical trials.

**Objectives:**

To explore the definitive evidence of mushroom on cognitive functions among at-risk middle-aged and young-olds.

**Design:**

The first study is a 10-year cognitive assessment follow-up on an existing cohort, the Diet and Healthy Aging (DaHA), which recruited over 1,000 older adults from the year 2010 to the year 2016. The second is a carefully designed randomized controlled trial (RCT) to assess the role of mushrooms in promoting cognitive functioning among around 600 middle-aged adults and young-olds.

**Participants:**

Participants were selected based on specific inclusion criteria, such as being community-living adults aged 45–74 years with a family history of dementia, APOE ε4 allele, or subjective cognitive decline, while consuming mushrooms no more than once a week and having no dementia, along with the exclusion of individuals with neurological or psychiatric disorders and significant sensory or motor impairments.

**Intervention:**

Participants in the intervention group will consume Pleurotus citrinopileatus mushroom powder daily for 24 months, with compliance monitored using electronic diary apps. The powder contains 7.0 mg/g of ergothioneine (dry weight).

**Measurements:**

Cognitive function including Mini Mental State Examination, Rey Auditory Verbal Learning Test, Symbol Digit Modality test, Clinical Dementia Rating etc. along with mental health and biological markers will be measured at baseline, 1 year, and 2 years after baseline.

## 1 Introduction

Cognitive declines can begin in one’s 30s, particularly affecting the ability to process new information and solve problems, though most people become aware of the decline in their 40s(Mitchell et al., 2023; Murman, 2015). Over time, this decline can become severe enough to lead to Mild Cognitive Impairment (MCI) and dementia. The latest nationwide epidemiological survey in Singapore reported a dementia prevalence of 10% among those aged 60 years and above (Subramaniam et al., 2015). This figure is expected to further rise in the coming years given the increasing numbers of the older adults in the population.

Maintaining cognitive function with advancing age is an important and challenging question that requires well-founded research with good translational opportunities. This is particularly important for at-risk populations such as Apolipoprotein E (*APOE*) ε4 allele carriers (Harris, 2024), those with a family history of dementia (Lee et al., 2022), and those at an early stage of cognitive decline, known as Subjective Cognitive Decline (SCD) (Jessen et al., 2014). Without early intervention, these at-risk populations groups are likely to progress to dementia. Prevention should be the focus at population level because current anti-dementia therapies only delay the progression of the disease. For example, the latest approved antibody intravenous (IV) infusion therapy, Lecanemab, only showed a moderately lesser decline in measures of cognition and function than placebo and it was associated with adverse events (van Dyck et al., 2023). Epidemiological studies have reported that higher mushroom consumption is associated with better cognitive performance—across word recall, executive function, and prospective memory—and with lower risk of incident dementia (Cha et al., 2024; Ba et al., 2022; Aoki et al., 2024). Mushrooms contain bioactive compounds such as ergothioneine, which has antioxidant and anti-inflammatory activity and has demonstrated neuroprotective effects in cell and animal models, but human evidence for a definitive neuroprotective effect remains limited. In a recent double-blind, placebo-controlled pilot trial, 14 older adults with MCI received ergothioneine supplementation (25 mg per capsule, thrice weekly) for one year. The intervention was well tolerated, and the ergothioneine group showed modest improvements on the Rey Auditory Verbal Learning Test, as well as stabilization of plasma neurofilament light chain levels—findings that suggest a potential slowing of neuronal damage but require confirmation in larger trials (Yau et al., 2024).

In Singapore, we found that participants who consumed mushrooms >2 portions per week had 47% reduced odds of having MCI (Feng et al., 2019). The association between mushroom consumption and MCI may be mediated by interleukin-6 and hypersensitive C-reactive protein (Chen et al., 2024). Those with higher mushroom consumption were also found to have better cognitive performance as assessed by the Digit Symbol Substitution test (DSST)(Ba et al., 2022; Yan et al., 2023; Nurk et al., 2010), Trail Making Test (TMT)(Yan et al., 2023), block design (Nurk et al., 2010), and the Word Learning test (CERAD-WL)(Ba et al., 2022). In an 18-year prospective study in the UK, mushroom consumers were found to have better cognitive performance than non-consumers across multiple cognitive domains. The relationship was observed to be dose-dependent, with those consuming one or more portions per week showing the highest cognitive score (Cha et al., 2024).

However, observational data do not prove causality, prompting five RCTs (3 weeks–6 months; *n* = 30–436), mostly using ergothioneine-rich Hericium erinaceus (HE), which have yielded mixed results: 3 g/day HE for 16 weeks modestly improved MCI cognition (reversed after a 4-week washout) (Mori et al., 2009), and 1.05 g/day for 49 weeks improved MMSE in AD patients (Saitsu et al., 2019), yet higher doses in healthy adults and a 436-subject, 24-week Australian trial showed no benefit (Zajac et al., 2020). Heterogeneity in species, cultivation, dosing, and duration complicates interpretation and generalization.

**Figure 1:**
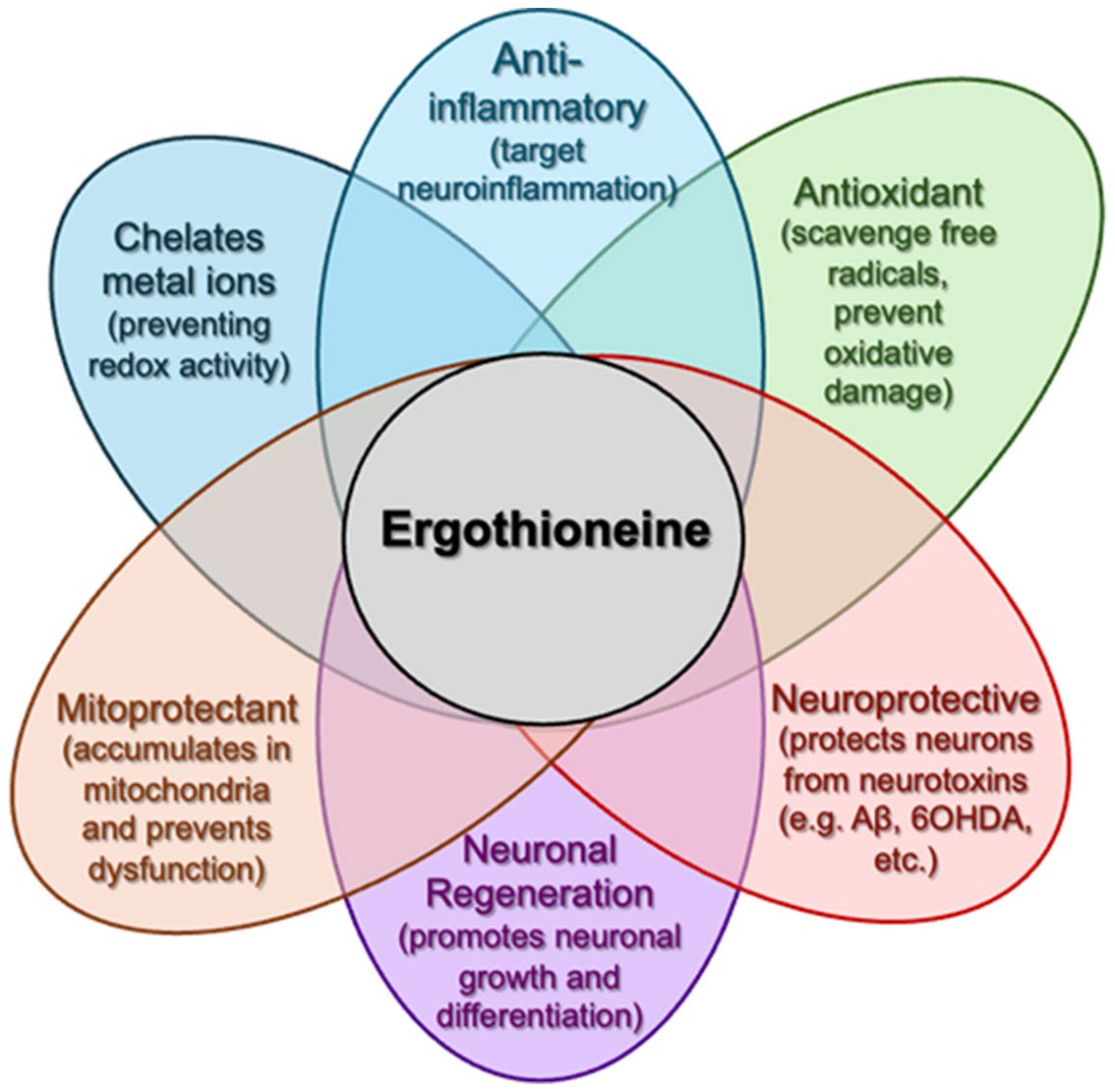
The multi-faceted capabilities of ergothioneine. Following oral administration, ergothioneine can accumulate in the brain (crossing the BBB) and has been demonstrated to counteract multiple pathologies (e.g., mitochondrial dysfunction, neurotoxin accumulation, neuroinflammation, etc.) associated with dementia and other neurodegenerative disorders.

Mushrooms contain bioactive compounds such as ergothioneine, which has demonstrated antioxidant and neuroprotective effects in cell and animal models (Cheah and Halliwell, 2021; Tang et al., 2018; Cheah and Halliwell, 2012; Wu et al., 2021; Cheah et al., 2016; Wu et al., 2022; Halliwell and Cheah, 2024). However, human data on ergothioneine’s cognitive benefits remain limited. Accordingly, our trial is designed to explore both clinical outcomes and underlying biological mechanisms—such as changes in plasma ergothioneine levels, inflammatory markers, and DNA methylation profiles—rather than to confirm a definitive neuroprotective effect (Figure. 1).

Due to a lack of definitive evidence of mushroom on both longitudinal relationships from prospective cohort studies and clinical efficacy from clinical trials, we propose two related sub-studies. The first sub-study is a 10-year cognitive assessment follow-up on an existing cohort, the Diet and Healthy Aging (DaHA), which recruited over 1,000 older adults from the year 2010 to the year 2016 (Yu et al., 2020). The long-term follow-up will provide further evidence to strengthen the conclusions from the proposed trial. The second sub-study is a carefully designed randomized controlled trial (RCT) to assess the role of mushrooms in promoting cognitive functioning among middle-aged adults and young-olds. With an adequate sample size and a long intervention period of 24 months, we will provide definitive data on the cognitive benefits of mushroom consumption.

## 2 Research aims and hypothesis

In this study, we aim to (1) assess the longitudinal relationship between mushroom consumption and cognitive decline, measured by the absolute change in Mini Mental State Examination (MMSE) scores over a median of 10 years among surviving DaHA participants; (2) test the effects of mushroom powder (two 500 mg capsules per day) on cognitive function measured using the Symbol Digit Modality test (SDMT), the Color Trail Test (CTT) and the Rey Auditory Verbal Learning Test (RAVLT) over 12 months and 24 months, in comparison with controls who consume placebo; (3) examine the underlying protective mechanisms using biomarkers of aging (epigenetic clocks and inflammation) and measuring blood ergothioneine levels; (4) explore epigenome-wide DNA methylation changes following mushroom powder intervention and assess whether such changes correlate with cognitive and biomarker outcomes, without presupposing a beneficial direction. We hypothesized that (1) mushroom consumption will predict slower cognitive decline in a dose-dependent manner over a median 10-year period among DaHA participants; (2) daily mushroom powder consumption over 12 and 24 months will improve cognitive function (processing speed, executive function, memory) among middle-aged and young-old adults; (3) daily mushroom powder consumption will lead to favorable changes in inflammatory marker of aging that correlate with the increase in bioactive compounds from mushrooms; (4) daily mushroom powder consumption will lead to changes in DNA methylation profiles.

## 3 Materials and methods

### 3.1 Observational study

We will conduct a 10-year follow-up on all survivors from DaHA study. The proposed follow-up will consist of a cognitive assessment session using the MMSE and the Clinical Dementia Rating (CDR) scale. Cognitive decline will be quantified as the absolute difference between baseline and 10-year follow-up MMSE total scores. The average decline rate will be calculated by dividing the change in MMSE total score by the time elapsed between baseline and follow-up in years. Cognitive impairment will be defined by a CDR score of 0.5 or above. We have published widely on aging related topics using data from the DaHA cohort (Feng et al., 2019; Ye et al., 2023; Li et al., 2019; Liu et al., 2021). As DNA methylation and inflammation markers have already been measured in over 600 samples from DaHA participants, further bio-sampling is not required. Details of the cohort can be found in our cohort profile paper (Yu et al., 2020).

### 3.2 Randomized controlled trial

We will conduct a randomized, controlled trial to assess the effects of mushroom powders on cognitive function and epigenetic and inflammatory markers of aging. There will be a total of 600 participants: 300 in the mushroom powder intervention arm and 300 in the placebo control arm. An interim assessment will be conducted when the sample size reaches 200 participants. Trial participants will be screened from two community-based observational cohort studies in Singapore [the DaHA (Chan et al., 2018) study and the Aging in a Community Environment Study (ACES) (Liew et al., 2018)], one completed RCT [the Choral Singing RCT (CSRCT)], and open recruitment via newspaper and poster advertisement at community centers, as well as advertisement on social media and websites. The above three studies led by the applicant have successfully recruited and assessed over 1,500 subjects. The mean age of DaHA, ACES and CSRCT participants in 2025 are projected to be 78, 80 and 79 years, respectively. Given the 20.7-year life expectancy at age 65 in Singapore. We estimate that at least 83% of them would be alive when the trial starts. All subjects have had comprehensive cognitive assessment and most of them have consented to be contacted for future research. Once eligible subjects from the two cohort studies are enrolled in the proposed RCT, they will no longer be considered part of the observational cohorts. No further follow-up visits will be conducted on these participants according to the DaHA-ACES cohort protocol, ensuring that the validity of findings from the cohort studies is not affected. This will be clearly communicated to trial participants selected from the cohorts. Our inclusion criteria are: (1) Community-living adult aged 45 years to 74 years. (2) Family history of Alzheimer’s disease or other types of dementia or carrying at least one *APOE* ε4 allele or having subjective cognitive (SCD) defined as “self-reported memory problems that have been getting worse over the past year.” (3) Consume mushrooms no more than once a week. (4) No dementia: CDR global score equal to zero. Exclusion criteria are: (1) Any diagnosis of neurological diseases (e.g., epilepsy, stroke) made by a clinician. (2) Any diagnosis of psychiatric illnesses (e.g., depressive disorders) made by a clinician. (3) Marked hearing impairment, visual impairment or upper limbs motor difficulties that make the person unable to complete cognitive assessment.

Details of the trial’s measurements and procedures will be explained to eligible participants in their native language. Written informed consent will be obtained only after potential participants are satisfied with their understanding of the trial.

### 3.3 Randomization

Trial participants will be randomly assigned to one of the two arms in an equal ratio using a web-based randomization system. Randomization will be performed by the Singapore Clinical Research Institute (SCRI) on a pay-for-service arrangement. The randomization sequence will be concealed from the investigators, ensuring allocation concealment.

#### 3.3.1 Standardization

**Figure 2:**
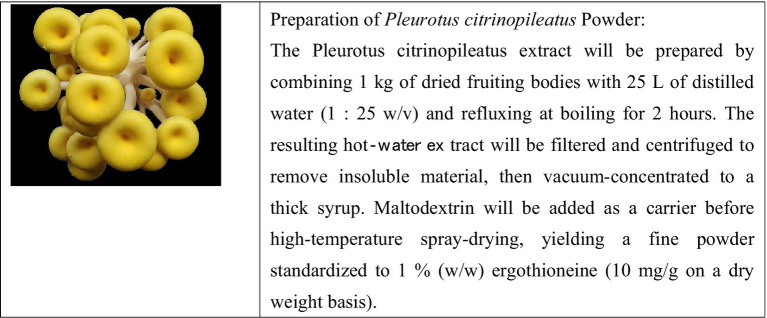
The diagram of the *Pleurotus citrinopileatus* body and its preparation method.

Participants randomized to the mushroom powder intervention arm will be instructed to consume daily for 24 months. Electronic diary apps will be used to monitor compliance. The Pleurotus citrinopileatus extract will be supplied by Guangdong Yuewei Edible Fungi Technology Co., Ltd. (Guangzhou, China). Dried fruiting bodies (1 kg) were mixed with distilled water (25 L), then boiled under reflux for 2 h. The slurry was filtered and centrifuged (4,000 rpm, 15 min) to yield the extract, which was vacuum-concentrated to 1/10 volume, blended with 20% maltodextrin, and spray-dried at high temperature. Final powder contained 1.0% ergothioneine (HPLC-UV quantification). We selected a daily ergothioneine dose of 10 mg (two 500 mg capsules of Pleurotus citrinopileatus extract at 1% ET), corresponding to 70 mg ET per week. This falls within the range of prior human interventional studies: Yau et al. administered 25 mg ergothioneine per capsule, three times per week (75 mg ET/week) for one year (Yau et al., 2024); Mori et al. gave four 250 mg tablets of Hericium erinaceus dry powder (96% purity, ~960 mg powder per dose) three times daily (~2.88 g powder/day) for 16 weeks—both regimens were well tolerated (Mori et al., 2009). Participants in the control arm will consume placebo capsules. Participants and assessors will be blinded to the randomization. The mushroom powder will be derived from the mushroom species *Pleurotus citrinopileatus*. The dried fruiting bodies of *Pleurotus citrinopileatus* have an ergothioneine content of 10.0 mg/g (dry weight). The following figure is a diagram of the *Pleurotus citrinopileatus* body and its preparation method (Figure 2).

### 3.4 Assessments and outcomes

Participants will undergo questionnaire-based interviews, cognitive testing, and clinical assessment. The primary endpoint is 24-month MMSE change; secondary endpoints include SDMT, CTT, RAVLT, CDR, plasma ergothioneine, inflammatory markers, NfL, and epigenetic age.

We will administer the Singapore–Chinese version of the MMSE to eliminate bias from items incongruent with Singapore’s geography and culture (e.g., seasons, state/province), thereby enhancing the accuracy and sensitivity of cognitive assessment in our local Chinese population(Feng et al., 2012), in which: (1) the seasons question is replaced by “Without looking at your watch, what time is it?”; (2) the city/town question is replaced by “What area are we in?”; (3) the state/province question is replaced by “Which part of Singapore is this place (North, South, East, West or Central)?”; (4) the three-item immediate and delayed recall words are adapted to “ball, flag, tree” (English) or “柠檬, 锁匙, 气球” (Chinese); and (5) the sentence repetition item is adapted to “no ifs, ands or buts” (English) or “四十四只石狮子” (Chinese). Trained study psychologists will administer RAVLT, Color Trails Test 1&2, and both written and oral versions of the SDMT to evaluate memory, executive function and information processing speed, respectively. The above neuropsychological tests have been used in local research studies by the Principal Investigator and colleagues (Feng et al., 2006; Feng et al., 2009; Lee et al., 2012). CDR (Morris, 1993) will be administered to assess dementia severity (CDR global score). Only participants with a CDR global score of zero will be recruited. Depressive symptoms will be measured using the 15-item Geriatric Depression Scale (GDS-15)(Sheikh and Yesavage, 1986) for older participants and Patient Health Questionnaire (PHQ-9) for middle-aged participants (Kroenke et al., 2001). The PHQ-9 is a short questionnaire that measures depression severity based on the DSM-5 criteria (Kroenke et al., 2001). The GDS has been used in local studies by the principal investigator and coworkers (Feng et al., 2012; Ng et al., 2009) Dietary intake will be assessed using a Food Frequency Questionnaire (FFQ)(Whitton et al., 2017) that the trial participants will complete at baseline and follow-up visits. Information on supplements that may have neuroprotective effects, such as curcumin/turmeric, *Gingko biloba*, and polyunsaturated fatty acids, will also be collected using a questionnaire that will be designed by trial dietitian. Venous blood specimens will be collected both for the measurement of biological markers and for liver and renal panel tests for safety monitoring. To ensure consistency and minimize the influence of other factors, biological sample will be collected in the morning between 8.15 to 9.30 a.m. following an overnight fast, both at baseline and 24 months post-baseline. Table 1 provides a summary of measurements and study visits schedule.

**Table 1:** Measurements and visit schedules.

S.no.	Measurement	Baseline	1 year	2 years
1	Socio-demographics	√		√
2	Lifestyles, dietary intake (FFQ)	√	√	√
3	Medical conditions, medications, supplements	√	√	√
4	Mini mental state examination (MMSE)	√	√	√
5	GDS-15 or PHQ-9 for depressive symptoms	√	√	√
6	Neuropsychological tests (3 cognitive tests as a battery)	√	√	√
7	Clinical dementia rating (CDR)	√	√	√
8	Handgrip strength, repeated chair rise test, blood pressure	√	√	√
9	Venous blood sample collection and blood-based biomarkers	√		√

For cognitive measures, we selected locally validated and normed tools to ensure sensitivity to changes over time. We have carefully considered and included a panel of biological markers based on potential pathways. We will measure pro-inflammatory cytokines (such as hs-CRP, TNFα, IL-1, IL-6, etc.), and neurofilament light chain (NfL) as a non-specific biomarker of neurodegeneration (AD). We will also measure the genetic polymorphism of *APOE* gene and the DNA methylation profile. This biological approach has a clear advantage over traditional trials that only assess clinical outcomes and clinical laboratory tests.

The level of inflammatory biomarkers and NfL in the blood will be assessed using an ELISA assays as previously described(Cheah et al., 2017). We decided to measure pro-inflammatory cytokines because both inflammatory damage has been well documented in both AD pathophysiology and the biology of aging(Querfurth and LaFerla, 2010) and the biology of aging(Franceschi et al., 2018). NfL levels have been suggested as a blood-based biomarker for neurological diseases (Fan et al., 2023). The level in plasma increases in preclinical Alzheimer’s disease (AD) before symptoms appear (Preische et al., 2019) and correlate with MCI and other AD hallmarks, such as brain shrinkage and tau tangles observed on PET scans (Raket et al., 2020). Here, we will use plasma NfL as a systemic biomarker for cognitive decline. We will generate epigenome-wide methylation data using the state-of-the-art Illumina Infinium MethylationEPIC BeadChip (Pidsley et al., 2016) on the HiScan system. The array covers over 850,000 methylation sites per sample at single-CpG site resolution, and provides unparalleled coverage of CpG islands, RefSeq genes, ENCODE open chromatin, ENCODE transcription factor binding sites, and FANTOM5 enhancers, as well as regions identified by the ENCODE project as potential enhancers. This epigenome approach will help shed light on unknown pathways and is superior to traditional approaches that focus on a limited number of genes. We will calculate epigenetic clocks using established methods and use these clocks as a quantitative measure of biological age compared with chronological age.

Furthermore, we will measure plasma ergothioneine levels using liquid chromatography-mass spectrometry at baseline, 12 months and 24 months. Correlating changes in blood ergothioneine levels with cognitive outcomes and with pathological and aging-related biological markers will shed light on ergothioneine’s mediating role in the hypothesized relationship between mushroom consumption and cognitive aging. The inclusion of biomarkers of both a mushroom-derived compound and of aging and dementia represents a novel approach not previously adopted in human studies.

### 3.5 Methods of analysis

To test the relationship between mushroom consumption and cognition among cohort participants: A linear regression model will be used to assess how baseline mushroom consumption predicts future cognitive changes. A log-binomial regression model will be used to compare the proportion of incidental cognitive impairment cases in the subgroups defined by mushroom consumption. A sensitivity analysis will be performed using a log-binomial model for repeated measures, including assessments at the 5-year follow-up and controlling for major demographic, medical, and lifestyle factors. Pre-specified subgroup analyses will stratify by baseline cognitive status (MMSE ≥ 27 vs. < 27), sex, and education level (≤ secondary vs. > secondary). Interaction terms will test for differential intervention effects.

*A priori* power calculation was performed using G*Power 3.1. Assuming a small-to-medium effect size of Cohen’s d = 0.30 in 24-month MMSE change, two-tailed *α* = 0.05 and 90% power, 235 subjects per arm are required. Allowing 20% attrition at 24 months (Jacobsen et al., 2021), we plan to enroll 300 per arm.

To test the effects of ET-Rich mushroom powder on cognitive function among RCT participants: A linear regression model will be performed to compare changes in cognitive scores at 24 months between the intervention and control arms. A sensitivity analysis will be performed using a general regression model for repeated measures, including assessments at 12 months and controlling for major demographic, medical and lifestyle factors at baseline.

To examine the underlying protective mechanisms: The levels of biomarkers will be treated as continuous variables. Changes in biomarkers will be calculated by comparing post-intervention levels to pre-intervention levels from intervention and control and represented in percentage (%). These changes will then be compared between the intervention and control groups. Furthermore, paired two-tailed t-tests will be performed to assess whether biomarker levels differ between pre-intervention and post-intervention measurements within each group (control and intervention). To analyze the ergothioneine data, we will use a “change-versus-change’ approach, using repeated ergothioneine measurements as predictors to examine their relationship with changes in cognition and other biomarkers. We will further employ Structural Equation Modeling (SEM) to assess how the intervention affects cognitive function, with ergothioneine as a hypothesized mediating factor. In the SEM, we will adjust for age, sex, education (social class proxy), BMI, APOE ε4 status, baseline supplement use (curcumin, Ginkgo, omega-3), individual metabolic variations by including plasma liver enzymes (ALT, AST) and baseline renal function & ergothioneine levels as covariates, and compliance rate. Interaction terms will test effect modification.

**Figure 3:**
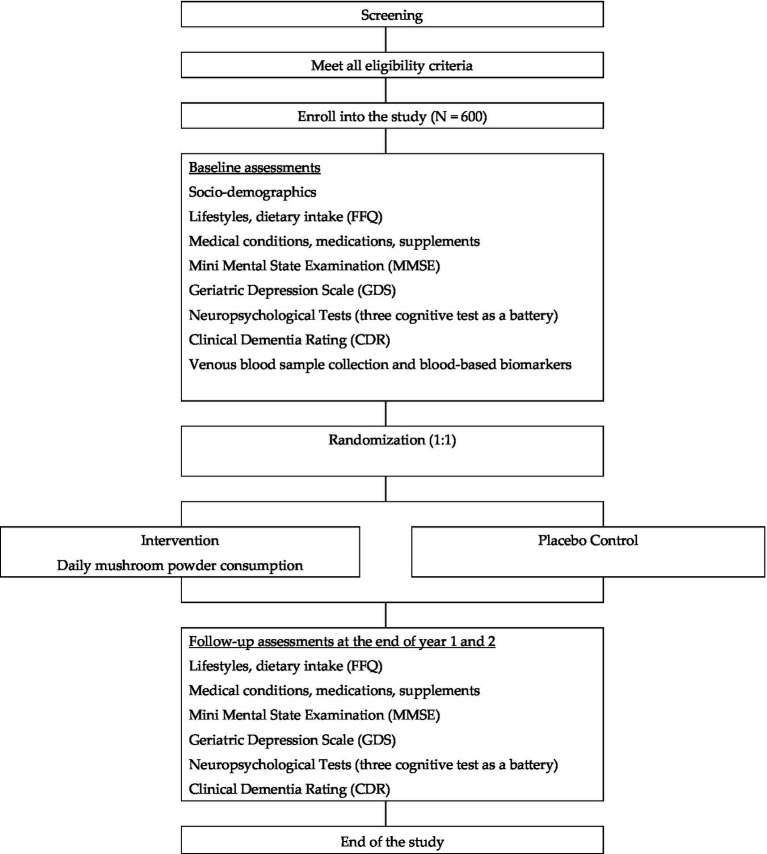
Flow diagram of study participants.

To examine the epigenetic changes associated with ET-Rich mushroom consumption: Analysis of epigenome-wide methylation data will be performed using a modified version of the CPACOR pipeline(Lehne et al., 2015). Briefly, we will normalize marker intensities by quantile normalization. Single-marker tests using logistic regression will be used to examine the association of mushroom consumption with each autosomal CpG site (yes/no), adjusted for age and sex. Principal components from intensity values of Infinium control probes will also be included as covariates in the regression models. Finally, the association results will be adjusted for the genomic control inflation factor. We will subsequently develop a methylation risk score (MRS) model by taking a weighted approach (by effect size) for the CpG loci identified to be associated with mushroom consumption. Figure 3 describes the flow of study participants.

### 3.6 Ethics statement

This study was carried out in accordance with the recommendations of the National University of Singapore (NUS) with written informed consent from all subjects. All subjects gave written informed consent in accordance with the NUS Human Biomedical Research guidelines.

### 3.7 Progress and next step

We have made significant progress in laying the foundation for our proposed studies. The DaHA study has successfully completed two waves of data collection. Wave 1 (2011–2017) recruited 1,010 community-living older adults aged 60 years and above in Singapore, collecting comprehensive data on lifestyle factors and cognitive function. Wave 2 (2017–2020) followed up with over 600 survivors (aged 62–91), providing valuable longitudinal data through established cognitive assessments. This rich dataset forms the basis for the planned 10-year cognitive assessment follow-up, which aims to further explore the longitudinal relationships between diet, nutrition, and cognitive health.

As for the next step, first, we will formally recommend that the Ministry of Health Singapore include mushroom consumption as a primary preventative measure in its Clinical Practice Guideline for Dementia. We will recommend that the Health Promotion Board Singapore include moderate mushroom consumption as a component in their public education and health communication programs that aim to promote healthy and functional aging. We will publish our findings in leading journals, present them at key international and local conferences, and share them with practitioners in Singapore and beyond. As a result, we anticipate that clinicians will recommend regular mushroom consumption to older adults consulting specialists or general practitioners for memory concerns. This recommendation will also extend to individuals at high risk of dementia due to family history or genetic testing, aiming for favorable clinical outcomes. Second, we will conduct further research to confirm long-term efficacy among RCT participants. We plan to conduct extended follow-up assessments 5 years post-baseline to assess clinical progression (defined as an increase in CDR global score from 0 to 0.5 or higher) and to identify incident cases of dementia in both the intervention and control groups. This extended follow-up study will be completed 3 years after the completion of the currently proposed study and will provide further evidence to strengthen the conclusions from the proposed trial. Last, the research team will seek potential public or private funding sources to form a spinoff/startup company focusing on the development of novel plant-based medical foods and nutraceutical products. The applicant and coworkers have been working on the cognitive benefits of various plant-based compounds. With a strong scientific foundation and a successful track record, we believe that establishing a spinoff/startup company specializing in phytoceuticals, nutraceuticals, and medical foods is highly feasible. This feasibility is supported by Singapore’s favorable biomedical R&D environment and potential funding from governmental bodies, venture capitalists, angel investors, and modern funding methods such as crowdfunding. The potential benefits for Singapore’s healthcare sector and economy are substantial and expansive.

## 4 Discussion

Mushroom consumption is safe and is likely to be embraced by older adults in the community as a preventive intervention. Although compliance to the intervention may raise as a potential problem given the long study period of two years, we have proposed the following methods to address this potential difficulty and limitation: (1) study staff to give weekly phone calls to remind study participants of the regime they are supposed to follow; (2) compliance will be monitored via a simplified one-touch electronic diary app (large icons, single-button “check-in”), augmented by automated push/SMS/phone reminders, caregiver assistance for entry, and cross-validation using electronic pill caps. Compliance rate can be used as a covariate in statistical analysis. Per-protocol analysis will be conducted on a subsample of subjects who have a compliance rate of 85% or above. Although we include a placebo control and blinding, repeated cognitive testing may introduce practice effects. We will account for this by including the assessment session (visit number) as a covariate in our longitudinal models, and where validated alternate forms exist, we will use them. With positive results and strong evidence, mushroom consumption and supplementation could be recommended as an important and simple method for promoting cognitive health in aging individuals and as a public health measure at the population level. This will potentially lead to healthcare cost savings. Furthermore, this study could provide evidence for the development of new nutraceutical products, with further translational research. The proposed study will provide more definitive data on the efficacy of mushrooms in preventing cognitive decline among individuals who are at high risk of dementia. Translating these findings into clinical and public health practice could lead to improved cognitive health and a reduced incidence of dementia. This, in turn, could result in substantial savings on the direct and indirect cost of dementia care in Singapore. The research will also provide insights into the biological mechanisms underlying the putative effect of mushroom powder on cognitive decline. By addressing scientific questions not previously rigorously examined, this research can strengthen our conclusions regarding clinical efficacy.
